# Altered sensory innervation and pain hypersensitivity in a model of young painful arthritic joints: short- and long-term effects

**DOI:** 10.1007/s00011-021-01450-5

**Published:** 2021-03-13

**Authors:** Luke La Hausse De Lalouviere, Oscar Morice, Maria Fitzgerald

**Affiliations:** Department of Neuroscience, Physiology and Pharmacology, University College London, Medawar Building, Gower Street, London, WC1E 6BT UK

**Keywords:** Inflammatory pain, JIA, Nociceptor, Pediatric, Adolescent, Joint afferents

## Abstract

**Background:**

Early life experience can cause long-term alterations in the nociceptive processes underlying chronic pain, but the consequences of early life arthritic joint inflammation upon the sensory innervation of the joint is not known. Here, we measure pain sensitivity and sensory innervation in a young, juvenile and adult rodent model of arthritic joints and test the consequences of joint inflammation in young animals upon adult arthritic pain and joint innervation.

**Methods:**

Unilateral ankle joint injections of complete Freund’s adjuvant (CFA) (6−20 µl) were performed in young, postnatal day (P)8, adolescent (P21) and adult (P40) rats. A separate cohort of animals were injected at P8, and again at P40. Hindpaw mechanical sensitivity was assessed using von Frey monofilaments (vF) for 10 days. Nerve fibres were counted in sections through the ankle joint immunostained for calcitonin gene-related peptide (CGRP) and neurofilament 200 kDa (NF200).

**Results:**

Ankle joint CFA injection increased capsular width at all ages. Significant mechanical pain hypersensitivity and increased number of joint CGRP + ve sensory fibres occurred in adolescent and adult, but not young, rats. Despite the lack of acute reaction, joint inflammation at a young age resulted in significantly increased pain hypersensitivity and CGRP^+^ fibre counts when the rats were re-inflamed as adults.

**Conclusions:**

Joint inflammation increases the sensory nociceptive innervation and induces acute pain hypersensitivity in juvenile and adult, but not in young rats. However, early life joint inflammation ‘primes’ the joint such that adult inflammatory pain behaviour and nociceptive nerve endings in the joint are significantly increased. Early life joint inflammation may be an important factor in the generation and maintenance of chronic arthritic pain.

## Background

A considerable number of children and adolescents suffer from joint pain associated with juvenile rheumatic disease; persistent pain is the most common and distressing symptom of juvenile idiopathic arthritis (JIA) [1]. Both clinical and experimental studies have highlighted the extent of this pain in young patients despite good disease control [1–5]. In the 5 years from disease onset, a subset of children with JIA (10–20%) develop persistent pain [4, 6–8] and there are indications that those with particularly poor pain outcomes might be identifiable at an earlier stage of the disease [6]. A wide range of factors appear to influence persistent pain in inflammatory joint disease [1, 9] but the measurable spread of this pain beyond diseased joints in both patients [3, 10, 11] and animal models [12] indicates an underlying biological mechanism of prolonged and widespread sensitization of pain circuitry within the central nervous system, driven by aberrant input from the joints or by central neuroimmune dysfunction [12–14].

The need for more research is evident but to date little is known about the signalling of pain from joints in young patients. In adult rodents aberrant nerve sprouting in arthritic joints is a contributor to pain related behaviour [15, 16]. The increased density of unmyelinated nociceptive C-fibres expressing calcitonin gene-related peptide (CGRP) and sympathetic fibres into areas of inflammation may contribute to persistence of pain when inflammation has subsided. CGRP expression in peripheral nerve cell bodies in the dorsal root ganglia following inflammation also increases and mirrors the pain behaviours seen in arthritic rats [16, 17]. Sprouting CGRP+ ve nerve fibres are found in the joints of arthritic humans and are closely associated with neovascularization of the joint following injury [18, 19]. It is not known whether sensory nerve sprouting occurs in inflamed joints of young rodents.

The normal development of somatosensory and pain processing in young mammals is dependent upon a balance of sensory information from skin, muscle and joints into the spinal cord in the first postnatal weeks [20–23]. The maturation process can be altered by abnormal patterns of afferent inputs arising from injured or inflamed tissues, and early life exposure to tissue inflammation leads to long-term changes in rodent somatosensory processing, pain transmission and analgesic responsiveness [13, 24]. A wide range of peripheral tissue injuries at critical development ages lead to functional changes in pain pathways that last into adulthood [25–28]. This includes experimental ankle joint inflammation in young rodents, which results in a distinct age-dependent neuroimmune reaction in the spinal cord followed by abnormally prolonged pain behaviour upon re-inflammation in later life [12]. It is not known whether aberrant innervation of the joint also contributes to this ‘priming’ of inflammatory pain sensitivity.

The aim of this study was to establish the role of joint innervation in acute and chronic joint inflammatory pain in young rodents. We have adapted the widely used model of single joint inflammation, intra-articular injection of complete Freund’s adjuvant (CFA) to animals of different ages [29]: postnatal day (P)8, P21 and P40 to model joint pain in the infant, child/adolescent and young adult [30]. The aims of the study were to compare the pattern of joint inflammation, sensory innervation and pain sensitivity at the three ages and to discover whether joint inflammation in early life, once resolved, can alter joint innervation and sensitivity to inflammatory in adulthood.

## Methods

### Animals

All experiments were performed in accordance with the United Kingdom Animal (Scientific Procedures) Act 1986. Reporting is based on the ARRIVE Guidelines for Reporting Animal Research developed by the National Centre for Replacement, Refinement and Reduction of Animals in Research, London, United Kingdom. Male Sprague–Dawley rats aged 8, 21 and 40 days (P8, P21, P40) were obtained from the UCL Biological Services Unit and maintained on a 12-h light/dark cycle at constant ambient temperature with access to food and water ad libitum. Pups were returned to their litter between testing; handling and maternal separation were kept to a minimum. All animals were bred and maintained in-house and exposed to the same standard caging, handling and diet throughout development. Table 1 provides details of the number of animals of each age used for each experiment.

**Table 1:** Number of animals used for behavioural pain testing, capsular width and immunohistochemistry in each group at each age

Experimental group	Naive		Single CFA		Repeat CFA	
	Rats	Joints	Rats	Joints	Rats	Joints
Behavioural pain testing						
P8	4	8	4	8	–	–
P21	3	6	3	6	–	–
P40	4	8	4	8	4	8
Immunohistochemistry and capsular width						
P8	4	8	4	8	–	–
P21	4	8	4	8	–	–
P40	4	8	4	8	4	8

Both ipsilateral (CFA treated) and contralateral ankles were used in each animal. The same joints were used for immunohistochemistry and measuring capsular width. See “Methods” section for power calculations

### Joint injections

Microinjection of commercially available complete Freund’s Adjuvant (CFA) (Sigma–Aldrich, UK) into the intra-articular space was used to produce acute arthritic inflammation of the right ankle joint (hock) under isoflurane anaesthesia (5% in 100% medical O_2_ for induction, 2–3% for injection). A 30-gauge needle attached to a 100 µl Hamilton Syringe was inserted into the right joint capsule from the posterior lateral aspect and advanced until a palpable release of pressure. The needle was then withdrawn slightly and the CFA injected. Following injection, the needle remained in the joint for at least 30 s to ensure that the CFA remained in the joint space. Young (P8, *n* = 4), adolescent (P21, *n* = 4) and adult (P40, *n* = 4) animals received 2 μl, 6 μl and 20 μl CFA, respectively. These volumes were determined empirically by injection of Evans blue into cadaveric ankles, with volumes that resulted in no spillage into the surrounding tissue. The repeat injection in adults was performed in the same manner as the initial injection, using 20 μl CFA in the ipsilateral joint.

### Behavioural pain testing

**Fig. 1:**
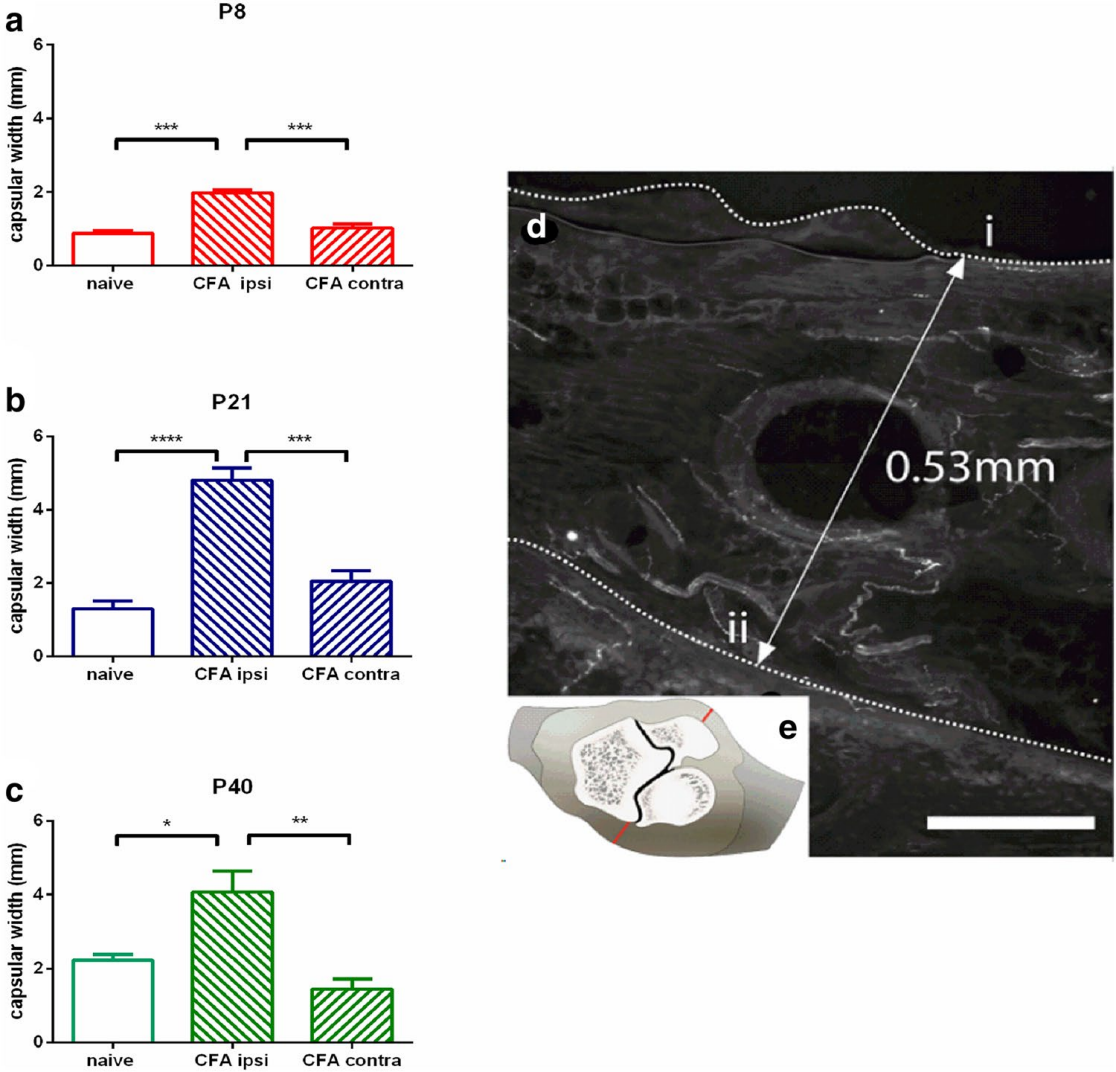
CFA-induced joint inflammation increases the capsular width throughout postnatal ages. Ankle capsular width measured on the injected site (ipsilateral), the opposite ankle (contralateral) and in untreated animals (naïve), in P8 (**a**), P21 (**b**) and P40 (**c**) animals, 4 days post-injection (**d**). Section through the ankle capsule to show width measured between the edge of the section (i) and the surface of the bone (ii) on both sides of the bone (**e**). Scale bar = 100 µm. One-way ANOVA, Tukey’s post hoc comparisons; *P < 0.05, **P < 0.01, ***P < 0.001, ****P < 0.0001

Hindpaw mechanical pain thresholds were established in naïve and CFA-injected young, postnatal (P)8, adolescent P21 and young adult P40, (*n* = 3–4 per group, see Table 1) rats using graded, calibrated von Frey (vF) filaments applied to the plantar paw. Adult (P40) and adolescent (P21) animals were placed in boxes with a wire mesh bottom to allow filament application to the plantar surface of the hind paw. VF filaments of increasing force were applied to the hindpaw. Each vF filament was applied six times at 1 s intervals. Nociceptive threshold was taken as the force that caused paw flick or hindlimb withdrawal on three or more occasions. In the young (P8) group, due to their size, animals were gently held whilst vF filaments were applied to the plantar skin on the infero-lateral part of the hind paw. Mechanical thresholds were assessed at baseline, prior to CFA injection, and 2, 4, 24 h and 2, 3, 7 and 10 days post-injection. All measurements were made within a 3-h window between 11 am and 2 pm, by a single experimenter. Mechanical thresholds across time and treatment group were compared using a repeat measures ANOVA with a Bonferroni post hoc test (Figs. 1, 4). Power calculations were performed by comparing mean ± SD of vF thresholds in control and CFA treated joints from published behavioral data [12] and revealed that three animals per group were sufficient, with a confidence interval (two-sided) of 95% and power of 80%.

### Decalcification

As bone mineralization increases with postnatal age, the optimal decalcification time was determined for different developmental ages to maintain the structural integrity of tissue sections. A protocol, generously provided by Dr. Patrick Mantyh (University of Arizona), was adapted for younger animals. Rats were terminally anaesthetized (150 mg/kg of sodium pentobarbital) and perfused transcardially with cold 0.1 M phosphate buffered saline (PBS—pH 7.4 at 4–8 °C) followed by a fixative (4% formaldehyde, 12.5% picric acid at pH 6.9). The ankles were removed on both sides and immersed for 24 h in the fixative at 4 °C, and then washed in 0.1 M PBS solution at 4 °C. Skin was then removed and the tissue placed in 10% ethylenediaminetetraacetic acid (EDTA) solution (pH 7.4 at 4 °C) for decalcification for a variable number of days: 3 days for young (P8) rats, 6 for adolescent (P21) rats and 20 for adult (P40) rats; EDTA was refreshed on a weekly basis. Once decalcification was complete, the joints were rinsed twice with 0.1 M PBS and stored in 30% sucrose solution for 72 h in a—20 °C freezer before 30 μm sections were cut and mounted onto slides (Superfrost Plus, VWR) for immunostaining.

### Immunohistochemistry

Tissue sections were cut through ankle joints to expose the capsule. Sections were cut in parallel onto slides such that each joint produced four representative sets of 40–50 sections. Two sets of 20 sections each (taken from the middle of the joint) were stained with antibodies against calcitonin gene-related peptide (CGRP; Sigma C8198, USA, 1:5000 anti-rabbit) and heavy-chain neurofilament (NF200; CH 22104, Neuromics, 1:5000, anti-chicken). Both ipsilateral and contralateral joints were analysed from each animal, with *n* = 4 per group.

### Capsular width and nerve bundle counting

Capsular width was measured as the distance from the bone to the edge of the fibrous joint capsule in the middle of each section (Figs. 1d, 3c). The number of animals used per group (*n* = 4) was indicated by power calculations, comparing published mean ± SD measures of edema measures in control and CFA treated joints [16], with a confidence interval (two-sided) of 95% and power of 80%.

The number of CGRP+ ve and NF200+ ve axon bundles was counted in a rectangular area defined by the width of the field of view down the microscope (Nikon Fluorescence) at the centre of the section (600 µm) in one direction and capsular width in the other. All nerve bundles were counted in the defined area, excluding fibres clearly associated with blood vessels. Counting was performed independently by two experimenters, who were blinded to the treatment group. The mean number of nerve bundles per section was calculated for the ipsilateral and contralateral joints for each animal. Thresholds were kept the same for counting nerve fibres in control and inflamed joints.

The mean number of nerve bundles per section was calculated for the ipsilateral and contralateral joints for each animal, with 20 sections analysed from each ankle. Capsular widths and the average number of nerve bundles were compared with a one-way ANOVA with Tukey’s post hoc test (Figs. 1, 3, 4). The number of animals used per group (*n* = 4) was indicated by power calculations, comparing published mean ± SD of CGRP+ve nerve density in control and CFA treated joints [15], with a confidence interval (two-sided) of 95% and power of 80%.

## Results

### Intra articular CFA induces localised joint inflammation at all postnatal ages

To assess the inflammation of the joint induced by the injection of complete Freund’s adjuvant (CFA), the capsular width was measured in tissue sections, 4 days post-injection (Fig. 1). In naïve animals, the capsular thickness increased with age from 0.79 ± 0.06 mm P8 to 1.5 ± 0.15 mm at P21, and 2.5 ± 0.2 mm at P40. At all ages, CFA injection resulted in a significant and comparable increase in the capsular width on the injected, ipsilateral side compared to naïve and contralateral ankles (Fig. 1a–c) (*n* = 4 rats, *n* = 8 per age, see Table 1). Naïve and contralateral CFA ankles had comparable capsular widths at all ages and experimental conditions.

### Age-related pain behaviour following an acute intra-articular inflammatory insult

**Fig. 2:**
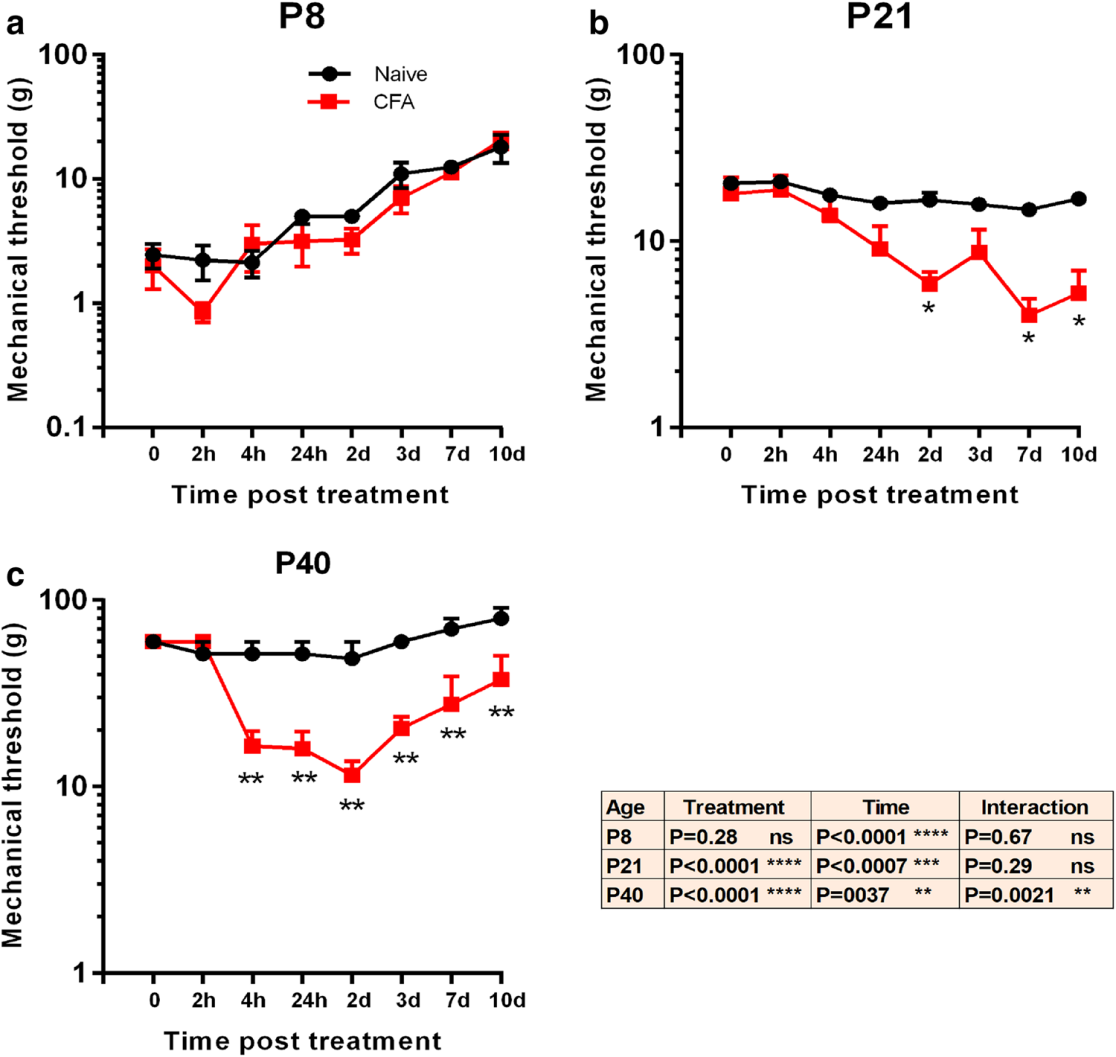
CFA-induced joint inflammation increases in mechanical sensitivity of the hindpaw at P21 and P40, but not P8. Hindpaw mechanical sensory reflex withdrawal thresholds were measured using von Frey hairs, before (time 0), and up to 10 days following CFA ankle joint injection in (**a**) P8, (**b**) P21 and (**c**) P40 animals. Mean mechanical thresholds in g.wt ± SE shown naïve (black) animals, and CFA treated (red) animals. Repeated measures ANOVA shows significant effect of treatment at P21 and P40 (*P* < 0.0001). Bonferroni post hoc test; *P < 0.05; **P < 0.01

Pain behaviour following CFA ankle injection was assessed for 10 days using the reflex withdrawal threshold evoked by punctate mechanical (von Frey filament) stimulation of the hindpaw (Fig. 2). As previously reported [31], young (P8) animals displayed a steady developmental increase in mechanical thresholds over the following 10 days of testing (P8 naïve at time 0, 1.7 ± 0.3 g, at time 10 days (P18) 18 ± 4.6 g, mean ± SE) (Fig. 2a), not seen in P21 and adult naïve animals (Fig. 2b, c). CFA injection into the ankle joint at P8 had no significant effect upon hindpaw mechanical thresholds (two-way ANOVA; treatment ns, see Table 1 for animal numbers, Fig. 2a). At P21, on the other hand, CFA significantly increased hindpaw mechanical sensitivity after the inflammatory insult peaking at 7 days and had not recovered at 10 days (two-way ANOVA; treatment *P* < 0.0001, Bonferroni post hoc comparison *P* < 0.05 at 2, 7 and 10 days, Fig. 2b). In P40 young adult rats the administration of CFA also significantly increased hindpaw mechanical sensitivity. Mechanical thresholds decreased from 4 h post-injection and remained low at 10 days (two-way ANOVA; treatment *P* < 0.0001, Bonferroni post hoc comparison *P* < 0.01 from 4 h to 10 days, Fig. 2c).

### Age-related changes in sensory innervation following acute arthritic joint inflammation

To quantify the effects of CFA inflammation on intra-articular innervation, sections through the ankles, taken 4 days post-injection, were immunostained for NF200, a marker of myelinated fibres and CGRP, identifying thinly myelinated and unmyelinated nociceptive nerve fibres. The number of immunostained nerve bundles were counted on the inflamed, contralateral and in naïve animals, in animals injected with CFA at P8 (young), P21 (adolescent) and P40 (adult).

**Fig. 3:**
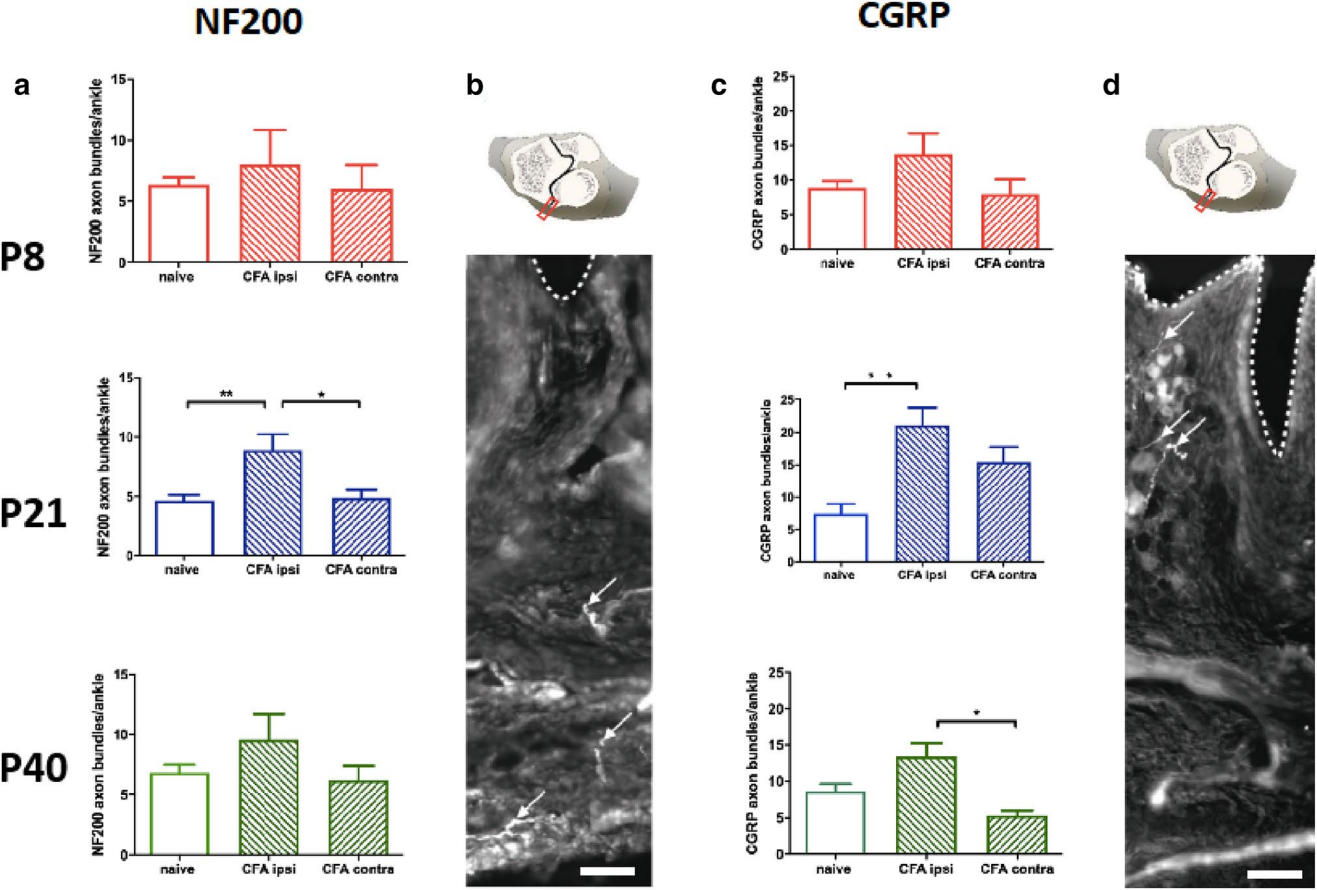
The effects of joint inflammation on NF200 and CGRP-positive nerve bundles. **a** Number of NF200- and **c** CGRP-positive nerve bundles in naïve, ipsilateral and contralateral ankle joints 4 days post-unilateral CFA injection in P8, P21 and P40 animals. One-way ANOVA, Tukey post hoc; *P < 0.05, **P < 0.01, ***P < 0.001. **b,** **d** Photomicrographs of sections identifying **b** NF200- and **c** CGRP-positive fibres for counting. The dashed line delineates the synovial membrane. Nerve bundles are marked with arrows. Scale bar = 100 µm. Above the photomicrographs are schematic representations of the strip of tissue, 600 µm across through the fibrous capsule, where nerve bundles were counted

Figure 3 shows that while the mean number CGRP+ve and NF200+ve nerve bundles within the capsule of naïve animals did not change with age, injection of CFA resulted in a significant increase in the mean number of nerve bundles per ankle in both adolescent (P21) and adult (P40) animals, when compared to the contralateral side or naïve animals, measured at 4 days post-injection. The mean number of CGRP+ve fibre bundles in CFA treated joints was increased at P21 compared to naïve joints (21 ± 2.7 versus 7.5 ± 1.5, *P* < 0.01, *n* = 4 per group, see Table 1) and at P40 when compared to the contralateral joints (13 ± 8 versus 5.3 ± 0.73, *P* < 0.05). The mean number of NF200 fibres significantly increased following CFA at P21, compared to both contralateral and naïve joints (8.9 ± 1.4 versus 4.6 ± 0.48, *P* < 0.01), but there was no effect at P40. No significant changes in nerve bundles was found following CFA injection at P8.

### Acute inflammation in young rat pups increases inflammatory joint pain and nerve sprouting in adults

The results above show acute arthritic ankle joint inflammation in young (P8) rats did not cause significant acute pain behaviour or change in sensory innervation, in marked contrast to the same inflammatory insult in adolescent and adult rats. To test whether ankle inflammation at P8 has longer term ‘priming’ effects, which would be evident when the animal reached adulthood, P8 animals were given a unilateral ankle joint CFA injection and returned to their litter to grow up. When they reached P40 the same joint was inflamed again (repeat CFA) and the effects compared to P40 animals receiving joint CFA for the first time (single CFA).

**Fig. 4:**
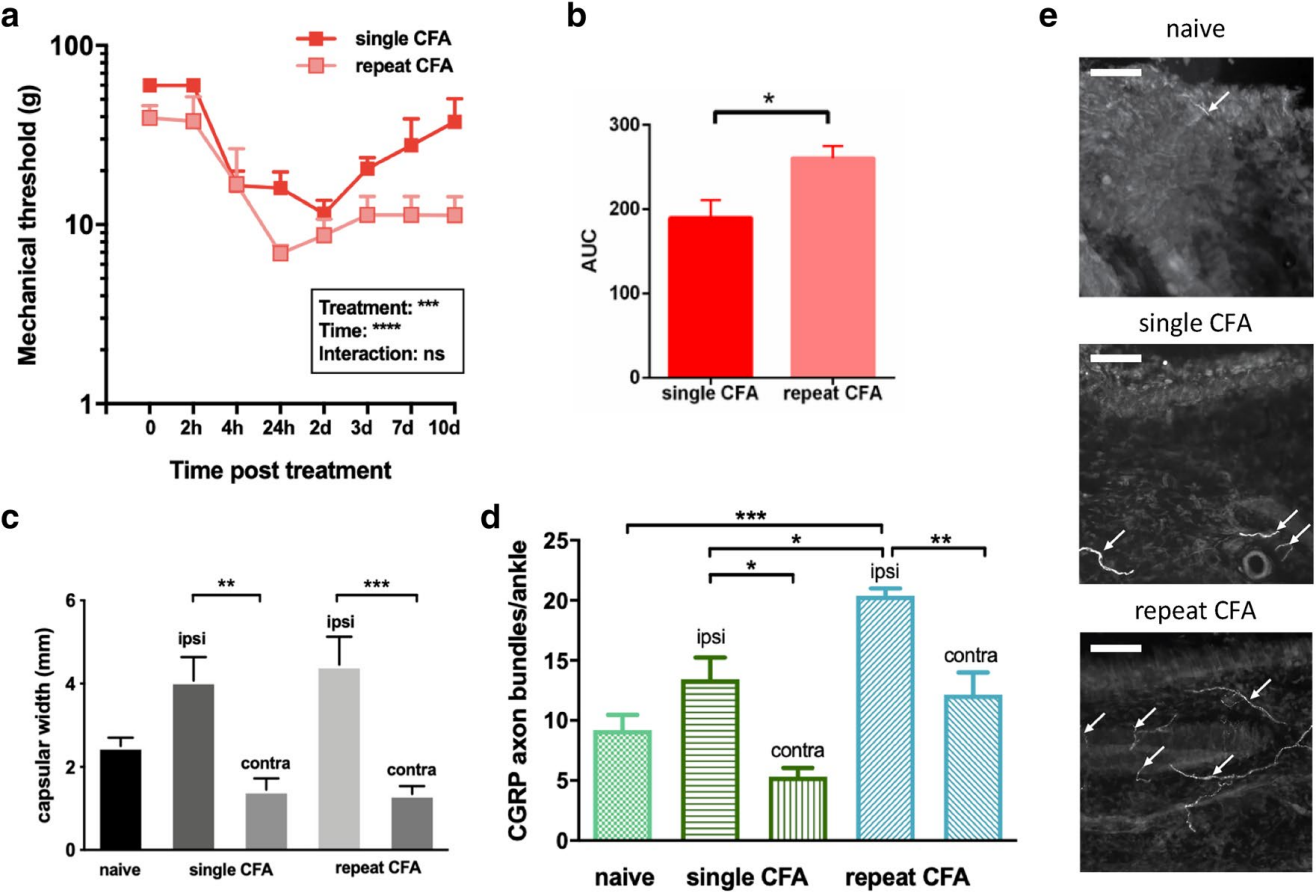
Early life joint inflammation ‘primes’ the pain response and sensory nerve sprouting to repeat inflammation in adults. Comparing data from P40 animals that received an ankle joint CFA injection at P8 and then again, in the same joint at P40 (repeat CFA) with P40 animals receiving joint CFA for the first time (single CFA). **a** Hindpaw mechanical sensory reflex withdrawal thresholds following single CFA (red) and repeat CFA (pink) (see Fig. 2 for details). **b** Area under the curve of threshold data, calculated relative to straight line baseline. **c** Changes in capsular width following single and repeat CFA (see Fig. 1 for details). **d** Mean number of CGRP + ve fibre bundles in the ankle joints following single and repeat CFA (see Fig. 3 for details). **e** Representative photomicrographs of CGRP-positive bundles in naïve, single and repeat CFA capsular tissue. The edge of the capsule is seen at the top of each image and arrows indicate positive fibres. Scale bar = 100 µm

Figure 4a shows that pain behaviour following CFA ankle injection, assessed for 10 days using the reflex withdrawal threshold evoked by punctate mechanical (von Frey filament) stimulation of the hindpaw was significantly enhanced in P40 rats whose ankle joint was inflamed when they were young, than those that are inflamed for the first time. The fall in hindlimb reflex withdrawal threshold in repeat CFA adults is significantly greater than that in animals inflamed for the first time (two-way ANOVA; treatment effect, *P* < 0.0001, df (7, 24), *n* = 4 for each group, see Table 1). The time course of the fall in threshold is different in the two groups. Figure 4b shows that the area under the curve for repeat CFA, calculated from a straight line baseline, is significantly greater than for single CFA at the same age, P40 (Unpaired *t* test, Welch’s correction, *P* = 0.037, *n* = 4 per group). This increased pain sensitivity occurs despite the fact that the ankle inflammation itself did not differ in the two groups. Figure 4c shows the capsular width 4 days post-CFA injection is not significantly different after single and repeat CFA (single 4.1 ± 0.6, repeat 4.5 ± 0.7, mean ± SE, *n* = 4, see Table 1).

Figure 4d shows that the mean number of CGRP axon bundles per adult ankle joint section, 4 days following repeat CFA, is significantly greater than that following single CFA at the same age (naïve 9.2 ± 1.3, single ipsilateral 13.4 ± 1.8, single contralateral 5.3 ± 0.7, repeat ipsilateral 20.4 ± 6.2, repeat contralateral 12.2 ± 1.8, (mean ± SE, *n* = 4, see Table 1). Repeat CFA in adult animals also resulted in an increased number of CGRP axon bundles in the contralateral, untreated ankle compared to the single CFA contralateral ankle (Fig. 4d). Thus, repeat inflamed ankles contained significantly more CGRP bundle fibres than single inflamed (*P < 0.05), naïve ankles (***P < 0.001) and the ankle contralateral to the repeat inflamed ankle (**P < 0.01). Repeat inflammation also results in enhanced innervation in the ankle contralateral compared to the single inflamed contralateral ankle (**P < 0.01), (One-way ANOVA with Tukey’s multiple comparison).

The mean number of NF200 axon bundles per section in the two adult (P40) groups, single and repeat CFA, were not significantly different (ipsi: 9.6 ± 2.1 v. 9.4 ± 1.4; contra: 6.2 ± 1.2 v. 5.7 ± 0.7).

## Discussion

This study used rodent models to compare arthritic joint inflammation pain behaviour and number of nociceptive nerve fibres in young, adolescent and adult rats and to test whether early joint inflammation has a long-term effect upon inflammatory pain and joint innervation in adults. Unilateral joint injection of complete Freund’s adjuvant (CFA) was an appropriate model as the focus here was on the inflammatory pain itself, rather than the arthritic disease process.

The results show that articular CFA injection caused significant and comparable joint swelling at all ages, indicating a robust local inflammatory response, independent of age. To measure the pain response to CFA, nociceptive thresholds on the paw were tested using von Frey (vF) filaments previously shown to reveal cutaneous pain sensitivity in young and adult rodents [31, 32]. The significant fall in the mechanical thresholds following joint CFA injections in adolescent and adult was still evident at 10 days post-injection and is consistent with earlier reports of pain sensitivity following CFA injection in adult rats [12, 29]. The data are consistent with the previous reports that full recovery of mechanical thresholds is reached in adult rodents at 12–14 days post-treatment whereas adolescent animals require 18–20 days [12].

The striking result here is that young, P8 animals did not develop significant mechanical hypersensitivity following CFA injection. As has been described elsewhere, hindpaw mechanical thresholds increase steadily over the test period [20] but the presence of ankle joint inflammation, clearly demonstrated by capsular swelling, had no effect upon the thresholds at any time following the CFA injection. Mechanical and thermal hypersensitivity has been observed as early as P0 following inflammation of the skin and subcutaneous tissue, although not as pronounced as those seen in older animals [33–35] so this absence of response must arise from the particular features of joint inflammation at this age. While it is possible that the swollen joint may have compromised movement or a classic display of hypersensitivity in young animals, it is more likely that spinal nociceptive withdrawal circuits are only weakly sensitized by afferent activity from the joint at this time. This is supported by the fact that dorsal horn neuron receptive field areas in P3 and P10 pups, already significantly larger than in adults, are unaffected by hindpaw inflammation, but are significantly increased by the same inflammation at P21 [36].

The lack of joint inflammatory mechanical sensitivity in young animals is paralleled by unchanged sensory innervation in the affected joint at this age. Several previous studies have reported that unilateral injection of CFA in adult results in sprouting of sensory nerve fibres in the affected joint [15, 16, 37]. Consistent with these previous reports, we observed significantly increased numbers of CGRP+ve fibres in the joint capsule accompanying the increased mechanical sensitivity in the hindpaw on the inflamed side in adolescent and adult rodents. The increase in CGRP nociceptor nerve bundles in adolescent and adult rats observed in this study is at an earlier time point (4 days) than previously demonstrated [15, 37], but otherwise similar. Importantly, we cannot conclude that these extra CGRP + fibres represent new nerve sprouts, which would require evidence of increased expression of growth associated protein GAP43 [38], rather than upregulation of CGRP within existing fibres, but in either case our data indicate a significant increase in local nociceptive signalling. It is accompanied by an increase in NF200 + fibres, a marker of larger myelinated afferents, commonly signalling non-noxious mechanical stimulation of the joints [39] at P21. CGRP+ve afferents are small and medium diameter nociceptors that respond to noxious stimuli and directly transmit signals to the spinal cord, and contribute to the increased synaptic strength between nociceptors and spinal neurons that underlies persistent pain [40] and activate neuronal transcription factors leading to the long-term changes in the neuronal excitability required to maintain chronic pain [41, 42]. These nociceptors also release CGRP from their peripheral terminals causing vasodilation and inflammation via CGRP1 receptors expressed by other sensory afferents, endothelial cells that line blood vessels, immune cells and keratinocytes [43]. Peripheral pharmacological inhibition of CGRPα peptide-receptor signalling selectively alleviates cutaneous inflammatory mechanical and heat hypersensitivity, demonstrating the importance of peripheral CGRP sensory terminals in inflammatory pain [44].

As activation of peripheral CGRP containing neurones are thought to play an important role in the genesis and maintenance of arthritic pain [45], the lack of change in CGRP+ nerve bundles in young inflamed joints may underlie the lack of behavioural hypersensitivity to joint inflammation in young animals. The lack of change is not due to an inherent inability of the young peripheral nervous system to respond to injury or inflammation in this way: both myelinated and unmyelinated sensory afferents sprouting follows cutaneous injury in young rodents leading to a hyperinnervation of the damaged and surrounding non-damaged skin, contributing to hypersensitivity around the wound [46]. In this case it is mediated by upregulation of the neurotrophin, NT3 in the skin following denervation [46], whereas increased innervation of inflamed joints is likely triggered by a range of factors released by immunocompetent cells, including NGF. NGF injection directly into joints is associated with synovitis and joint pain and blocking NGF action in the skin or joint has been used in human and rodent models to reduce pain [15, 47]. Sprouting CGRP expressing neurones also co-express the low affinity nerve growth factor (p75NGFR) supporting a role of NGF in increased innervation of joints following inflammation [48] and this role of NGF is already functional in the youngest of rodent pups [49].

Despite the absence of acute pain and altered sensory innervation following joint inflammation at P8, this treatment does increase inflammatory mechanical pain sensitivity and joint innervation to repeat inflammation in adult animals, compared to adults experiencing joint inflammation for the first time. Thus, joint inflammation in young animals appears to have a ‘priming’ effect, leading to an enhanced response to inflammatory pain in adulthood. This is first time that such priming has been reported from early life joint inflammation, but it has some similarities to that reported for other types of inflammatory injury in early life: such as neonatal hindpaw plantar incision [25, 31], hindpaw inflammation with agents such as carrageenan (CAR) or complete Freund’s adjuvant (CFA) [26, 34, 50, 51], full thickness skin wound [46], repeated needle prick [27, 52] and visceral injury caused by distension or inflammation [53, 54]. The mechanisms underlying this long-term priming are not known but there is evidence that priming of microglia in nociceptive circuits are required, at least in males [25, 55]. The results presented here showing increased inflammation induced CGRP+ nociceptive afferents following early life priming, suggests that peripheral macrophages may also be involved in long-term priming. Pain priming following P8 CFA inflammation may also be due to a developmental shift in the spinal cord neuroimmune profile. Following nerve injury in young animals, the ‘default’ neuroimmune response in the spinal cord is skewed in an anti-inflammatory direction, suppressing the excitation of dorsal horn neurons and preventing the onset of pain [56]. In this case, pain hypersensitivity emerges later in life, when the animal reaches adolescence (P21) and the neuroimmune profile shifts in a pro-inflammatory direction, unmasking a ‘latent’ pain response to an earlier nerve injury [32]. Recent studies demonstrate that the cytokine profile in the spinal cord following joint CFA changes with age, with prolonged upregulation of spinal IL6 at P21, but not P40 rats [12].

Here, we have extended a well-characterised animal model of joint inflammatory pain into childhood and adolescence and demonstrated the importance of taking an age-appropriate approach to understanding joint pain. The results are relevant to the re-inflammation pain of adolescent and adult patients with JIA [13] as it is well-documented that many JIA patients suffer from re-inflammation in adulthood [57]. Furthermore, while many factors, such as age, [8–11] sex, [8, 12, 13] events [4], and dysfunctional health beliefs [15, 16] have all been implicated as possible contributors to prolonged pain in adolescent JIA patients, our data suggest that while joint inflammation in childhood may not be very painful, re-inflammation of the joint at a later stage in life, will result in greater hyperinnervation and greater pain. Furthermore, we suggest that a better understanding of the mechanisms underlying joint hyperinnervation and the potential for priming in early life, may facilitate more targeted treatment of arthritis related pain.

The limitations of this study include the time point for measurement of joint innervation which was restricted to 4 days post-CFA injection to reduce animal use. The time course of this increased innervation at each age and in the primed adults would add to our understanding of the relationship between innervation density and pain behaviour. Furthermore, although previous studies have shown that CFA ankle inflammation has subsided by 10 days post-injection [12], we cannot assume that the re-inflamed ankle was completely normal before the second injection. The interpretation could be improved if we had a group injected with CFA at P8 and then vehicle at P40 to indicate how much of the increase in nerve bundles is due to prior events versus the second CFA injection. A wider range of pain measures beyond mechanical reflex threshold would also be useful, although young animals are not able to perform more complex tasks such as weight-bearing and conditioned place preference, reliably. The study is restricted to male rats which is a limitation as evidence for sex differences in inflammatory pain mechanisms is increasing [58].

## Conclusions

Joint inflammation induces increased sensory nociceptive CGRP+ ve innervation and acute pain hypersensitivity in juvenile and adult, but not in young rats. However, early life joint inflammation ‘primes’ pain processes in young animals such that when adult, nociceptive innervation and pain behaviour following joint inflammation are significantly increased. The results suggest that early life joint inflammation is an important factor in the generation and maintenance of chronic arthritic pain.

## Data Availability

Please contact the corresponding author.

## Abbreviations

P: Postnatal day
CGRP: Calcitonin gene-related peptide
NF200: Neurofilament 200
CFA: Complete Freund’s adjuvant
vFh: Von Frey hair
